# Activation and Stabilization of Lipase B from *Candida antarctica* by Immobilization on Polymer Brushes with Optimized Surface Structure

**DOI:** 10.1007/s12010-022-03913-9

**Published:** 2022-03-31

**Authors:** Dennis Sebastian Wunschik, André Lorenz, Kim Nadine Ingenbosch, Jochen Stefan Gutmann, Kerstin Hoffmann-Jacobsen

**Affiliations:** 1 Chemistry Department, Institute for Coatings and Surface Chemistry, Niederrhein University of Applied Sciences, Adlerstr. 32, 47798 Krefeld, Germany; 2 Deutsches Textilforschungszentrum Nord-West gGmbH, Adlerstr. 1, 47798 Krefeld, Germany; 3 Physical Chemistry and CENIDE (Center for Nanointegration), University Duisburg-Essen, Universitätsstraße 5, 45117 Essen, Germany

**Keywords:** Enzyme immobilization, Lipase, Polymer brushes, Non-native media, Biocatalysis

## Abstract

A reusable support system for the immobilization of lipases is developed using hybrid polymer-inorganic core shell nanoparticles. The biocatalyst core consists of a silica nanoparticle. PMMA is grafted from the nanoparticle as polymer brush via ARGET ATRP (activator regenerated by electron transfer atom transfer radical polymerization), which allows defining the surface properties by chemical synthesis conditions. Lipase B from *Candida antarctica* is immobilized on the hybrid particles. The activity and stability of the biocatalyst are analyzed by spectroscopic activity analysis. It is shown that the hydrophobic PMMA brushes provide an activating surface for the lipase giving a higher specific activity than the enzyme in solution. Varying the surface structure from disordered to ordered polymer brushes reveals that the reusability of the biocatalyst is more effectively optimized by the surface structure than by the introduction of crosslinking with glutaraldehyde (GDA). The developed immobilization system is highly suitable for biocatalysis in non-native media which is shown by a transesterification assay in isopropyl alcohol and an esterification reaction in n-heptane.

## Introduction

Enzyme catalysis has become an indispensable tool of organic synthesis in academia and industrial application, due to the intrinsically “green” nature of enzymatic biocatalysts, their high selectivity, and the mild reaction conditions provided [1–3]. The application of enzyme catalysis has been significantly extended from the physiological reactions to a broad variety of reaction types and substrates due to enzyme promiscuity [4–6] as well as protein engineering [6, 7]. Here, organic solvents play a crucial role, enabling new reactions in the absence of water as well as the conversion of water insoluble reactants. Yet, the presence of organic solvents often strongly impedes enzyme activity, stability, and the solubility of the catalyst itself [8, 9], a problem that is typically overcome by enzyme immobilization [10]. This technique includes the physical or chemical binding of an enzyme in or onto a support/matrix to generate a heterogenous system [11, 12]. Immobilization usually results in increased stability and reusability of the biocatalyst, but reduced enzyme activity. In principal, three basic methods of enzyme immobilization can be distinguished [13], entrapment [14, 15], adsorption [16, 17], and crosslinking [18, 19], but hundreds of methods based on combinations thereof have been developed. Although altered selectivity and enhanced activity by immobilization have been reported sporadically [13, 14], enzyme immobilization is a mostly empirical technique, where the optimal catalyst is typically found by experimental design. A rational design of the solid biocatalyst design by surface properties has been suggested before [20], but remains an objective of research to be achieved.

Lipases have emerged as one of the leading biocatalysts with a broad scope of applications in chemo-enzymatic synthesis ranging from enantioselective reactions via polymer synthesis to biodiesel production [21, 22]. Lipases (EC 3.1.1.3.) belong to the hydrolysis family and show the physiological function of ester hydrolysis [23] in aqueous systems, whereas the inverse reaction and esterification, as well as transesterification and amidation, are feasible in non-native media [24, 25]. Lipase B from *Candida antarctica* (CalB) is the most widely used lipase to date due to its high resistance to solvents and temperatures and the broad substrate spectrum [26]. In enzymatic synthesis, CalB is typically applied as solid biocatalyst after immobilization on polymeric microporous beads. The support of the commercial biocatalysts Novozyme 435^®^ and CalB Immoplus^®^ consists of poly(methyl methacrylate) (PMMA) which is crosslinked with divinyl benzene [27]. Polymeric beads are available in a broad range of surface properties. Yet, these polymeric materials suffer from low solvent resistance and low mechanical stability.

Polymer brushes have gained a great deal of attention in material sciences due to the tunability of surface morphology, energy and chemical functionality, and the ability to switch these properties by external stimuli [28–31]. Currently, the application of these properties in various fields such as hydrogels as well as sensors is under investigation. In this context, the application of polymer brushes in enzyme immobilization has attracted some interest [20, 32]. Polymer brushes are very promising materials for a rational design of enzyme supports for biocatalysis as well as for sensing applications [33, 34]. Recently, different lipases have been immobilized on silica microparticles modified with poly(sulfobetaine methacrylate)/poly(poly(ethylene glycol)methacrylate) brushes. An increase in temperature stability with respect to the enzymes in solution was found for a variety of lipases, whereas CalB activity was slightly disturbed by the presence of the polymeric brushes. These differences were explained by the different surface energies of the lipases under investigation [35, 36]. Yet, no silica-supported brushes of PMMA, which is ubiquitely used as carrier resins for lipase immobilization in biocatalysis, have been explored before.

In the present study, CalB is immobilized on spherical silica nanoparticles which are functionalized with a polymethyl methacrylate (PMMA) brush shell. The polymer brushes are prepared by atom transfer radical polymerization (ATRP) allowing a precise control of the brush properties by synthesis conditions. The interdependency of the surface properties and biocatalyst performance is analyzed and the surface parameters driving enzyme performance on the surface are analyzed. The applicability and reusability of the heterogeneous lipase catalyst for biocatalytic synthesis are analyzed with a special emphasis on activity and stability in non-aqueous environment and compared to a commercial microporous bead product in order to elucidate the difference of polymer brush versus microporous bead carriers.

## Experimental

### Materials

Lipase B form *Candida antarctica* (lipozyme CalB) was a kind gift from Novozymes (Bagsværde, Denmark). CalB immo Plus was purchased from c-LEcta, Germany. Copper bromide, N,N,N′,N″,N″-pentamethyldiethylenetriamine, methyl methacrylate (MMA), 4-methylumbelliferyl butyrate, oleic acid (88%), and tetramethyl orthosilicate were purchased from Sigma Aldrich/Merck (Darmstadt, Germany)). 3-Aminopropyltriethoxysilane and 3-(trimethoxysilyl)propyl 2-bromo-2-methylpropanoate were from abcr (Karlsruhe, Germany). Acetonitrile, ascorbic acid, anisole, ethanol (abs.), glutaraldehyde (25%), isopropyl alcohol, methanol, n-hexane, tetrahydrofuran (THF), and pyridine were from Carl Roth (Karlsruhe, Germany). MMA was purified using an aluminum oxide column followed by distillation before polymerization and isopropyl alcohol was dried with molecular sieves. All other chemicals were used as received.

### Particle Synthesis

Silica particles were generated using 2.8 ml (19 mmol) tetramethyl orthosilicate (TEOS) and 2.8 ml (34 mmol) ammonia (25%) dissolved in 35 ml ethanol abs. The reaction was performed at room temperature for 24 h. The sol-gel was washed three times with 10 ml ethanol and resuspended in 10 ml ethanol. Silanization was performed with a 1% (v/v) silane solution in ethanol consisting of different ratios of 3-aminopropyltriethoxysilane (APTES) and 3-(trimethoxysilyl)propyl 2-bromo-2-methylpropanoate (AIS). Surface functionalization took place at 60 °C for 24 h. The particles were washed three times with 10 ml ethanol and dried under argon.

For surface polymerization, 9.8 ml (92 mmol) of freshly purified MMA was dissolved in 9.3 ml anisole, degassed three times by a freeze-pump-cycle, and added to 800 mg dried particles under argon. Polymerization was performed with 5.14 mg (0.023 mmol) CuBr_2_ and 48.5 µl (0.230 mmol) PMDETA for 1 h at 50 °C under argon atmosphere. The functionalized particles were isolated by centrifugation and washed several times with the following series of solvents, THF, then THF/ddH_2_O (50/50), ddH_2_O, acetonitrile, and methanol, and finally dried with compressed air.

### Particle Characterization

Scanning electron microscope (SEM) images were acquired with a S-3400 N II microscope from Hitachi High-Technologies Europe GmbH. The particles were homogenized in 40 ml ddH_2_O using an ultrasonic homogenizer prior to analysis.

Thermogravimetric analysis was performed under nitrogen atmosphere with a TG 209 F1 Libra instrument (Netzsch, Germany) using a temperature ramp from 100 to 980 °C at 20°K/min. The mass loss was determined between 100 and 850 °C.

Gel permeation chromatography was performed with a GPC Viscotek 270 max (Malvern Instruments, UK) equipped with two T6000M 300 × 8 mm columns at 35 °C and a differential refractive index as well as UV detector. Tetrahydrofuran (THF) was used as eluent. Molecular weights were calculated based on poly(methyl methacrylate) standards. In order to analyze the molecular weight of the polymer brushes, a small amount of free initiator was added to the monomer solution before polymerization. The polymers synthesized in solution were precipitated in methanol and then recrystallized twice by dissolving in chloroform and precipitating in methanol. The purified PMMA was analyzed by gel permeation chromatography as reference for the polymer brushes synthesized simultaneously.

The dynamic light scattering (DLS) experiments of polymer grafted nanoparticles were performed at 25 °C on a Zetasizer Nano ZS particle analyzer (Malvern Instruments, UK) using a detection angle of 173° and a 4 mW He-Ne laser operating at 633 nm. All particle samples were homogenized in ddH_2_O by ultrasonification for 20 min on ice before DLS measurements. The sample cell was located in the middle of a temperature-controlled vat with toluene as the index matching fluid.

Water contact angles were measured with an OCA 15plus contact angle goniometer (dataphysics, Germany). Therefore, polymer was grafted from the surface of clean coverslips (High Precision, 18 × 18 mm, No. 1.5 H, Marienfeld, Germany) via the same procedure as described for the silica particles.

### Enzyme Immobilization and Biocatalyst Characterization

Immobilization of CalB was carried out in 10 mM phosphate buffer pH 8.0 at 20 °C for 24 h. One hundred fifty milligrams of modified particles was homogenized in 22.5 ml buffer rsp. with 2% glutaraldehyde (GDA). Of lipozyme CalB, 2.5 ml was added as received. The biocatalyst was washed with 50 ml buffer and 10 ml isopropyl alcohol and dried at room temperature.

Activity of the immobilized as well as native lipase B from *Candida antarctica* was analyzed by a fluorescence hydrolysis assay using the fluorogenic ester 4-methylumbelliferyl (4-MU) butyrate as described previously [37]. Briefly, the initial rate of 4-MU hydrolysis with 100 µM substrate and 10 mg dried particles was determined in 10 mM phosphate buffer (pH 8.0) with a Varian Cary Eclipse spectrometer and used as activity measure.

The transesterification activity of 10 mg CalB/PMMA/silica particles was analyzed with 100 µM 4-MU-butyrate in dried (using molecular sieve) isopropyl alcohol over 6 reaction cycles at 25 °C. The excitation wavelength was 324 nm and the emission at 382 nm was detected using a detector voltage of 600 V.

The esterification activity was analyzed with an absorption spectroscopy assay as described before [38, 39]. Therefore, a stock solution, consisting of 15.87 ml (50 mmol) oleic acid dissolved in 1 l n-heptane, was freshly prepared before use. Two milliliters of the stock solution (including 0.1 mmol oleic acid) and 15.9 µl (0.2 mmol) ethanol were added into a round bottom flask and the reaction was started by adding 10 mg CalB/PMMA/silica particles. The esterification was performed at 45 °C under continuous mixing. After 24 h, the reaction was quenched by addition of 0.5 ml HCl (14%) and the phases were separated. Afterwards, the organic phase was diluted in 1.2 ml fresh n-heptane and 1 ml copper acetate (50 g/l, pH of 6.1) was added. The amount of esterified oleic acid was determined from the vortexed solution via absorption at 705 nm using a UV-1650 PC spectrometer (Shimadzu, Japan).

## Results

### Surface Modification and Characterization

**Fig. 1:**
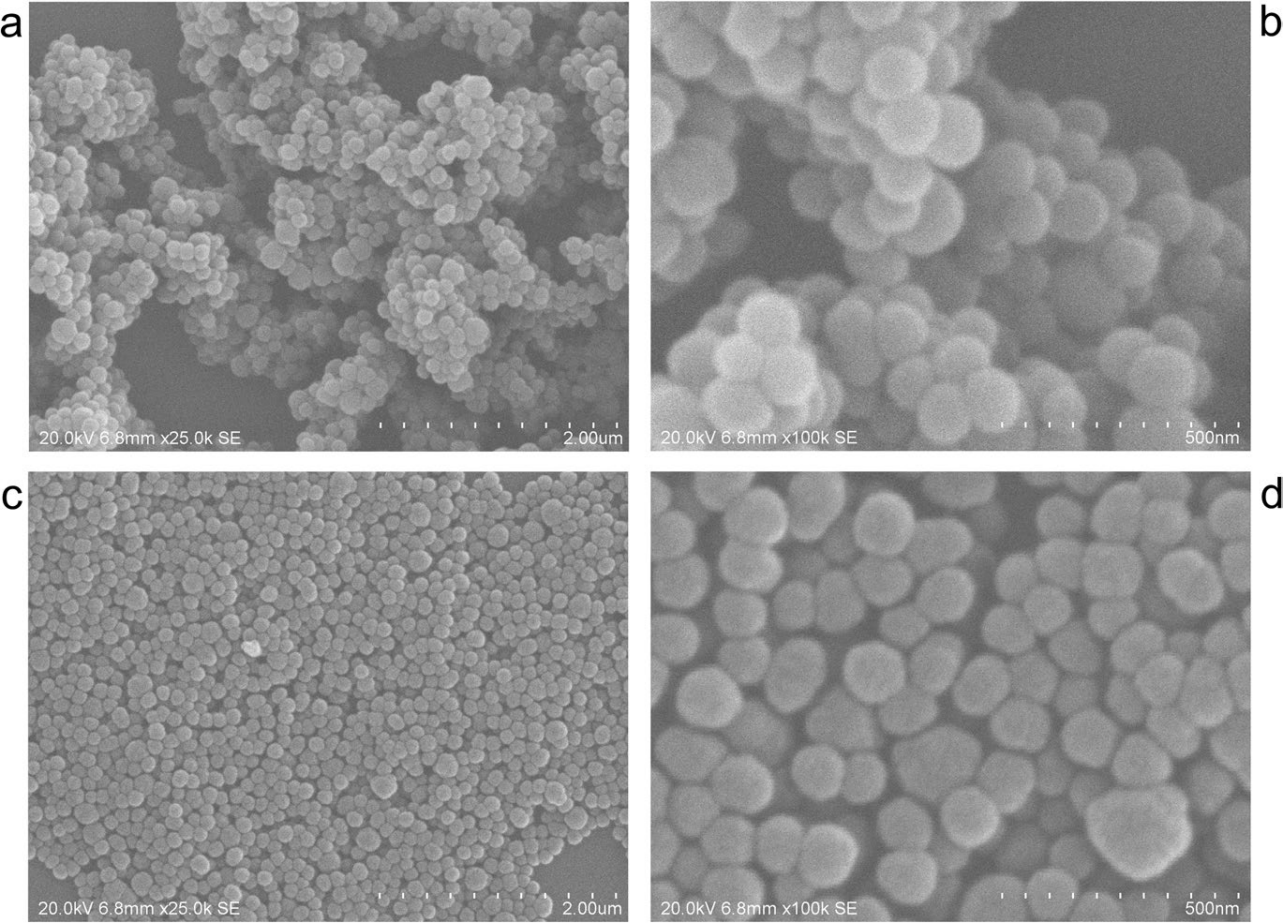
Scanning electron microscope images of pure TEOS particles (**a**, **b**) and PMMA-coated TEOS particles with 100% AIS functionalization at a magnification of 25k (**a**, **c**) and 100k (**b**, **d**)

**Fig. 2:**
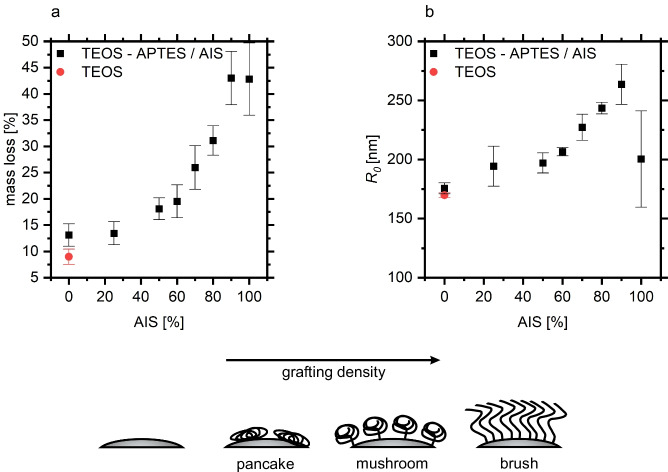
Thermogravimetric analysis (**a**) and dynamic light scattering measurements (**b**) of PMMA-modified TEOS particles with different grafting densities as regulated by the relative concentration of the initiator silane AIS and APTES in the silanization solution

Polymer brushes were grown on spherical silica particles synthesized by a Stöber process. In the Stöber synthesis, silica particles were grown to sizes in the range from 100 to 200 nm, as determined by scanning electron microscope (SEM) analysis. Figure 1a and b show the respective SEM images of the particles. The particle size was confirmed by dynamic light scattering analysis (Fig. 2), where a hydrodynamic radius of 170 ± 5 nm was found.

Surface modification with PMMA brushes was performed using ATRP grafting from technique. The SEM images of the PMMA-coated particles (Fig. 1c and d) showed uniformly coated particles. The molecular weight of PMMA brushes was estimated by GPC analysis of the free polymer formed concurrently in solution to 31,000 ± 600 g*mol^−1^.

The surface structure was varied via the polymer grafting density at constant polymer length by regulating the amount of surface bound initiator. For this purpose, solutions with different molar ratios of initiator silane (AIS) to amino silane (APTES) concentrations were applied to the silica particles. The polymer load of the particles was quantified by thermogravimetric analysis (Fig. 2a) revealing a non-linear increase in grafting density with increased initiator silane concentration. This is in line with a structural transition of the polymer layer from intermediate pancake and mushroom conformations to upright polymer brushes in the region from 50 to 70% AIS [40]. As depicted in Fig. 2b, dynamic light scattering (DLS) analysis corroborated this interpretation revealing sharply increasing hydrodynamic radii beyond 70% AIS. The large error bar of the hydrodynamic radius at 100% AIS was assigned to concurrent side reactions at a very high AIS load on the surface.

This analysis confirmed that the grafting density could be tuned with the percentage of AIS. Thus, the influence of the grafting density and hence surface structure on the activity of immobilized CalB was investigated in the following.

### Catalytic Performance of CalB Immobilized on PMMA-Coated Silica Particles

**Fig. 3:**
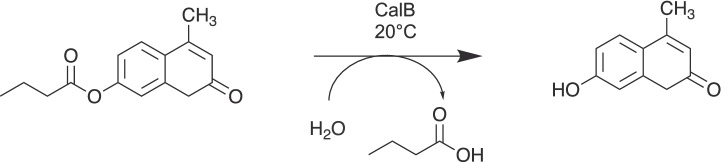
Lipase catalyzed hydrolysis of 4-MU-butyrate

Lipase B from *Candida antarctica* (CalB) was immobilized on silica/PMMA particles with different grafting densities. The effectiveness of the carrier was analyzed in terms of initial enzyme activity as well as reusability of the heterogeneous biocatalyst by a fluorescence hydrolysis assay using 4-MU-butyrate as substrate (Fig. 3).

**Fig. 4:**
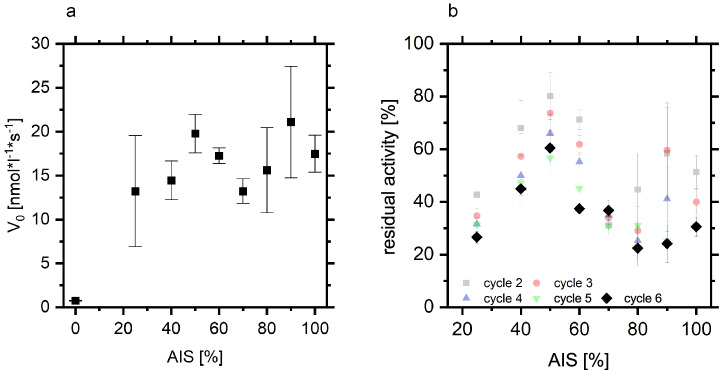
Activity (*v*_0_) of 10 mg biocatalyst as a function of AIS functionality (**a**). Activity retention depicted as residual activity after 2–6 reaction cycles with respect to the initial activity in the first cycle (**b**)

Figure 4a demonstrates the activity boost resulting from polymer coating of the silica particles with respect to silane modification. Silanization is a common technique for surface modification in different protein immobilization strategies [41, 42]. While nearly zero activity was observed after immobilization of the lipase on the silanized surface already, the presence of a small amount of PMMA on the silica surface induced lipase activity. Interestingly, local activity maxima at intermediate AIS fraction (ca. 50%) and 80–100% AIS were observed. A local activity minimum was found in between these maxima. The reusability of the biocatalysts was analyzed via the activity retention after 6 reaction cycles. As depicted in Fig. 4b, a clear maximum of reusability of the immobilized lipase was found at 50% AIS concentration coinciding with the local activity maximum.

The lower reusability of biocatalysts prepared from polymer brushes with very low grafting densities indicates that these surfaces provide minimal protection against desorption. Very low grafting densities lead to a sparse polymer layer on the surface (s. cartoon, Fig. 2). Here, the lipase molecules interact with the aminated surface and the unstructured polymer resulting in weak primary adsorption (on the silanized silica surface) and secondary adsorption (“on top” of the polymer layer). At intermediate grafting densities, polymer structures of limited order cover the silica surface which offer void volumes for tertiary adsorption of enzymes into the polymer structure. The activity minimum of the biocatalyst is found at the grafting densities where the chains begin to behave as brushes. This suggests that polymer brushes provide less attractive interaction sites for lipase adsorption than the mushroom structure. Hence, a high polymer density is required to facilitate lipase adsorption. Notably, these dense structures are not favorable for irreversible enzyme immobilization. These results are consistent with the analysis of laccase adsorption on 2-(dimethylamino)ethyl methacrylate where a maximum laccase activity was found at intermediate grafting densities [43]. We suggest that the activity and reusability maxima at intermediate AIS fractions arise from two counteracting effects: (a) a polymer structure and density required to shield the lipase from the unfavored silica surface and (b) the mushroom to brush transition which hampers lipase penetration into the polymer structure. Hence, the surrounding irregular polymer structure at intermediate AIS fractions inhibits lipase desorption processes and biocatalytic activity is retained.

The enzyme load of the polymer grafted silica particles was calculated from the activity difference of the immobilization solution before and after immobilization. With the enzyme load, the specific activity of the adsorbed enzyme (activity/enzyme) could be determined from the global activity based on the total weight of the biocatalyst (activity/g_catalyst_). As depicted in Table 1, the specific activity of CalB increased by a factor of 10 upon adsorption to the PMMA brushes at room temperature. In contrast, a decrease by a factor of 10 of CalB activity has been observed on poly(ethylene glycol) methacrylate (PEGMA) and sulfobetaine methacrylate (SBMA) brushes at ambient temperature whereas these surfaces led to activity boosting of other lipases [35]. This shows that the PMMA surface is exceptionally suited for CalB immobilization.

Due to the large experimental error of the determination of the enzyme load, the PMMA brushes with different grafting densities lead to identical adsorbed CalB activities within the statistical error of the experiment (Table 1). The water contact angles of the respective PMMA brushes prepared on planar glass surfaces were used to assess the surface energy. No significant differences were observed with different AIS/APTES ratios (Table 1) and the obtained value was close to the contact angle of PMMA polymer (67°). This emphasizes that the differences in biocatalyst activity at different grafting densities result from the different structures and not from different surface energies.

As depicted in Fig. 4, the activity data acquired from lipase adsorbed on polymer brushes with high densities showed a large scatter. This agrees with the low reusability of the respective biocatalyst. Weakly bound CalB is prone to leaching and leads to a broader distribution of the adsorption frequency. Hence, PMMA brushes with 50% AIS provide the optimal surface for CalB immobilization, considering the adsorbed amount, specific enzyme activity, and irreversibility of immobilization.

**Table 1:** Enzyme load and specific hydrolysis activity of CalB on the brush surface

Enzyme formulation	Enzyme load/mg·g^−1^	Specific activity/l^−1^·s^−1^	Contact angle/°
Solution	-	0.082 ± 0.006	-
PMMA (50%-AIS)	22 ± 6	0.75 ± 0.22	60 ± 1
PMMA (80%-AIS)	16 ± 5	0.80 ± 0.32	62 ± 1

Specific activities were calculated with the enzyme load and the data given in Fig. 4 and compared to the activity in solution. The contact angle depicts the water contact angle of the respective polymer brush prepared on a planar glass coverslip surface

**Fig. 5:**
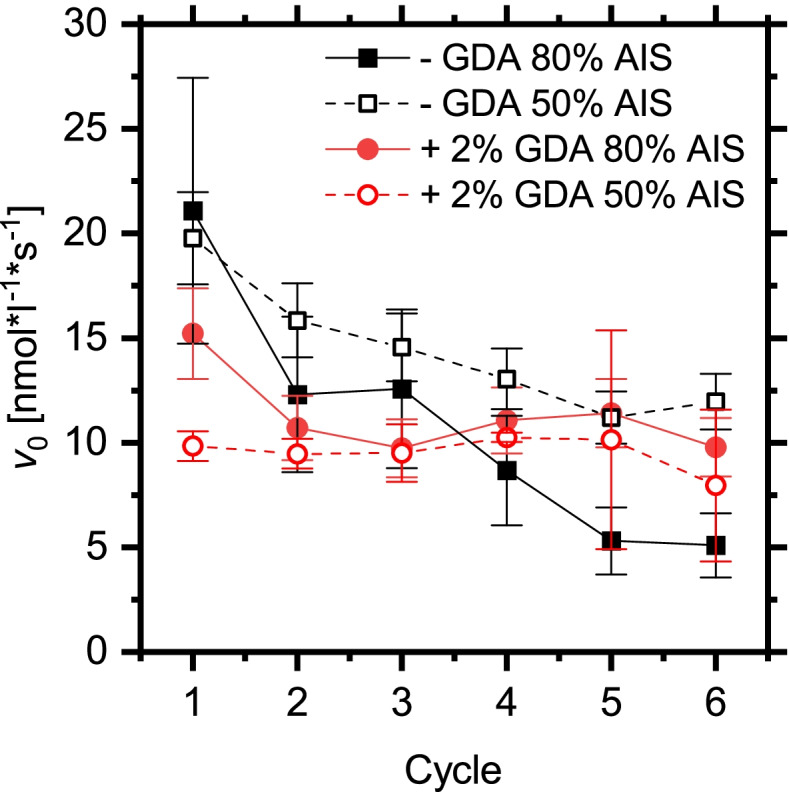
Hydrolysis activity of CalB on silica/PMMA particles with 50% and 80% AIS functionality with and without glutaraldehyde (GDA) crosslinking in repetitive reaction cycles

Irreversible protein immobilization can be forced by covalent crosslinking of the enzyme. The effect of covalent crosslinking was analyzed via the addition of glutardialdehyde to the immobilization process. As depicted in Fig. 5, the initial activity of the biocatalysts decreases upon crosslinking. Crosslinked enzymes typically lose activity due to restricted mobility and accessibility of the enzyme but gain stability [44]. The stability of the biocatalyst was enhanced by crosslinking as indicated by the lower slope of the activity loss with reaction cycle (Fig. 5). However, the final activity after six reaction cycles could not significantly outperform the optimum adsorptive bound lipase biocatalyst (50% AIS). Interestingly, the activity of the biocatalyst with intermediate grafting densities is more strongly impeded by crosslinking than the biocatalyst with higher polymer density. This is in accordance with an entrapment of lipase in the disordered polymer structure which is less favored in the case of crosslinked lipases. In the presence of GDA, the lipase can also be covalently attached to the aminosilane on the surface which produces a stable lipase immobilization with low reactivity.

### Activity in Non-native Media

**Fig. 6:**
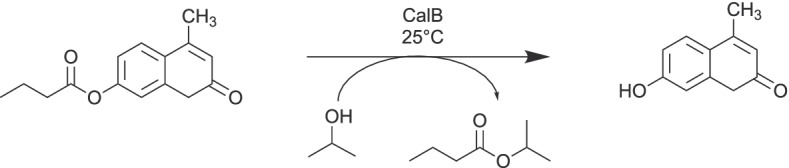
Reaction scheme of the transesterification of 4-MU-butyrate with isopropyl alcohol

For a potential application in chemical industry, the activity and stability of heterogeneous enzyme biocatalysts are of major importance, as especially a major part of lipase catalyzed reactions have to be performed in the absence of water [2, 3, 45]. For the analysis of its applicability in organic solvents, the silica/PMMA particles (50% AIS) with adsorptive bound CalB were used for the transesterification reaction of 4-MU-butyrate. A successful transesterification was conducted in isopropyl alcohol, where the alcohol served as solvent and substrate (Fig. 6). The commercial performance reference CalB immo Plus showed a 3.5-fold higher biocatalyst activity per weight than CalB on silica/PMMA particles whereas the specific activity of CalB was ca. 1.5-fold higher on the PMMA/silica biocatalyst (Table 2). It must be noted that the enzyme load of CalB immo Plus was estimated to 10% w/w according to the manufacturer’s information.

**Table 2:** Transesterification activity of immobilized CalB on PMMA-coated particles (50% AIS) in comparison to the reference CalB Immo Plus as analyzed by 4-MU release according to Fig. 6

Biocatalyst	Biocatalyst activity (µmol*l^−1^*s^−1^)*g^−1^_(carrier)_	Specific activity (µmol*l^−1^*s^−1^*g^−1^_(enzyme)_)
CalB immo Plus	6.97 ± 0.47	70 ± 5
PMMA (50%-AIS)/silica	2.18 ± 0.04	101 ± 2

**Fig. 7:**
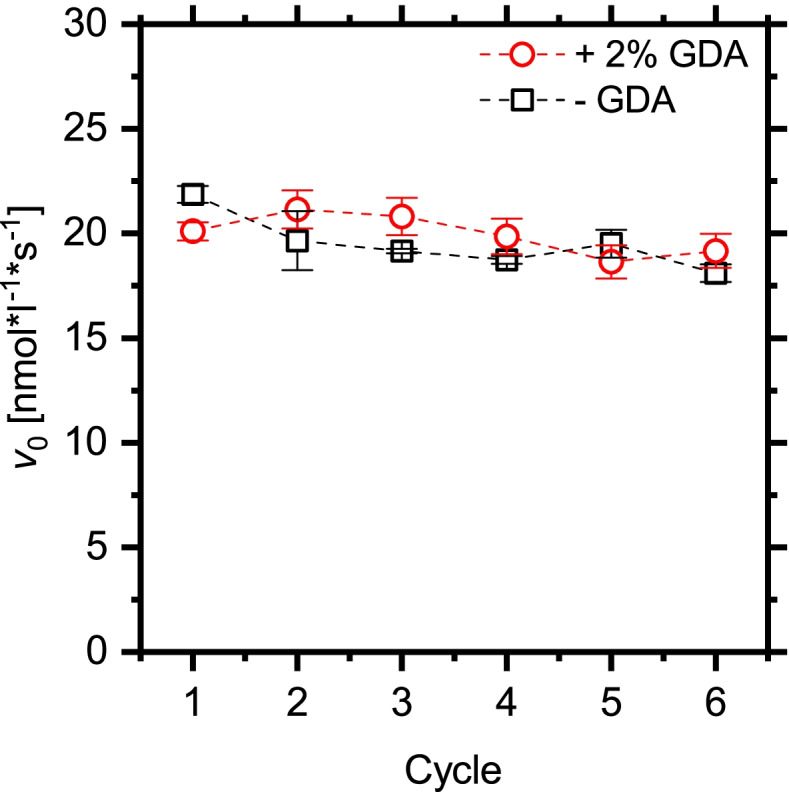
Biocatalyst activity and reusability in isopropyl alcohol as analyzed by 4-MU-butyrate transesterification of 10 mg heterogeneous biocatalyst over six reaction cycles

As depicted in Fig. 7, the biocatalyst could be used in six successive reaction cycles. In contrast to the reaction cycles in aqueous solution (Fig. 5), the silica/PMMA biocatalyst showed no significant loss of activity over these reaction cycles. The performance was not altered by crosslinking with GDA.

**Fig. 8:**
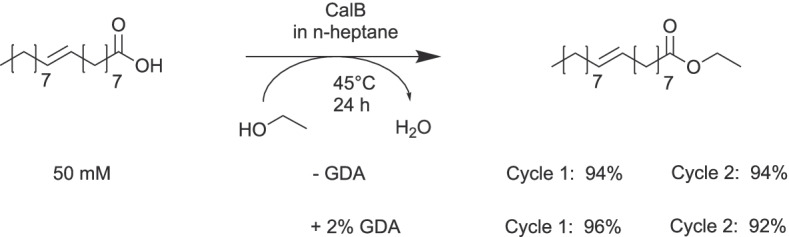
Esterification of oleic acid with ethanol in n-heptane with repeated usage of the biocatalyst

In order to analyze the biocatalyst applicability in a non-polar solvent, the esterification of oleic acid with ethanol was performed in n-heptane (Fig. 8). The formation of the ethyl oleate was analyzed by the spectrophotometric detection of residual oleic acid via its copper complex [39]. After 24 h, a complete conversion of oleic acid was achieved with CalB on silica/PMMA particles, a value highly competitive with other immobilization strategies [46]. In a reference experiment, we obtained the same conversion with 5 mg CalB immo Plus^®^. Moreover, the hybrid biocatalyst could successfully be reused in the same reaction under activity retention regardless of additional GDA crosslinking. n-Heptane is a non-polar solvent (logP = 4.27) which is commonly better tolerated by lipases than polar solvents with intermediate logP values as isopropyl alcohol (logP = 0.074) [47, 48]. Our analysis shows that the adsorption on the PMMA brush surface (50% AIS) is sufficient to irreversibly immobilize CalB and to retain its activity over a number of reaction cycles in organic solvents of diverse polarities. We suggest that this is a direct result of the decreased energy of solvation of the lipase in organic solvents leading to an intensified lipase attraction to the PMMA surface.

## Discussion

The prepared PMMA brushes on silica nanoparticles provide a balanced surface with a surface structure that can be tuned by the grafting density in the synthesis process. The high specific activity of immobilized CalB reveals that the intermediate surface energy of PMMA [49] is not only attractive to CalB but also hyperactivating the enzyme. It is already known that among many different protocols for immobilization of lipases, the use of hydrophobic supports is a very efficient method. Here, interfacial activation by opening the amphiphilic lid which covers the active site of many lipases [50, 51] via hydrophobic interactions of the lipase with the surface of the carrier material plays a significant role [52]. Different hydrophobic supports have been used to this purpose, like octyl-agarose beads [53], decaoctyl sepabeads [54], silica beads coated with acyl groups, polypropylene [55], or styrene/styrene divinylbenzene beads [17, 56]. The activation of CalB on well-defined modified silica surfaces has been discussed previously in the context of silanized silica surfaces and [57] SBMA/PEGMA polymer brushes [36]. It has been suggested that hydrophobic surfaces lead to a more open conformation of CalB giving easier access to the substrate [57]. Yet, in a more generalized computational analysis, it was suggested that the optimum support for individual lipases can be predicted using the free surface energy of solvation as predictor and the mechanism driving hyperactivation exceeds the nature of the lid [36]. Interestingly, CalB showed the highest free energy of solvation of the lipases under investigation which represents a high hydrophobicity. Our data is in line with this theoretical framework showing that the more hydrophobic polymer PMMA with a contact angle of 67° is more favorable for CalB activation than the polymers used in the study by Sánchez-Morán et al. with contact angles ranging from 5 to 58°. However, the activity maximum at 50% AIS found in this study cannot be explained by the surface energy.

Polymer brushes on silica surfaces do not only allow tuning the surface energy by choice of the monomer composition but also regulating the structure of the surface. Our data emphasize the importance of tertiary adsorption of the lipase into the polymer structure for the stability and reusability of the biocatalyst. Remarkably, the problem of enzyme leaching is more pronounced in aqueous environment. This is in line with a change of the free energy of solvation which is supposedly shifted towards positive values in organic media, especially in non-polar solvents.

The silica core PMMA brush shell particles offer an additional advantage for biocatalytic applications in organic media in comparison to polymeric beads: The biocatalyst can be applied long term even in good solvents for PMMA without physical disintegration of the biocatalyst. Whereas the polymeric beads are slowly dissolved in good solvents, the PMMA brushes are covalently linked to the insoluble inorganic support. Hence, the polymer adopts the swollen state allowing substrate transport to the tertiary adsorbed enzyme, but it stays intact. In order to develop a biocatalyst with maximum activity, optimizing the enzyme load will be a major challenge which can be addressed by tuning brush thickness [58].

## Conclusions

We report the successful immobilization of CalB on PMMA brushes grafted from spherical silica nanoparticles inducing enzyme hyperactivation on the surface. The hyperaivation is assigned to the low surface energy giving rise to an activating interaction of the surface with the hydrophobic patches of CalB. The PMMA brush structure dominates the stability of the biocatalyst in terms of reusability in multiple reaction cycles, which cannot be outperformed by covalent immobilization. The hybrid biocatalyst can be successfully applied in organic solvents of varying polarity. Thus, we present a new methodology for the immobilization of lipase B from *Candida antarctica* on inorganic core/organic shell particles leading to high activity, stability, and reusability in aqueous and non-native media media of the enzyme, which will be specifically interesting for applications involving good solvents for PMMA.

## Data Availability

The datasets generated during and/or analyzed during the current study are available from the corresponding author on reasonable request.
